# Harnessing Arsenic Derivatives and Natural Agents for Enhanced Glioblastoma Therapy

**DOI:** 10.3390/cells13242138

**Published:** 2024-12-23

**Authors:** Bo Yuan, Hidetomo Kikuchi

**Affiliations:** 1 Laboratory of Pharmacology, Graduate School of Pharmaceutical Sciences, Josai University, Keyakidai, Sakado 350-0295, Saitama, Japan; 2 Laboratory of Pharmacotherapy, Graduate School of Pharmaceutical Sciences, Josai University, Keyakidai, Sakado 350-0295, Saitama, Japan; hkikuchi@josai.ac.jp

**Keywords:** glioblastoma, temozolomide, arsenic trioxide, arsenic disulfide, darinaparsin, anthocyanidins, tetrandrine, bufadienolides, combination therapy

## Abstract

Glioblastoma (GBM) is the most common and lethal intracranial tumor in adults. Despite advances in the understanding of the molecular events responsible for disease development and progression, survival rates and mortality statistics for GBM patients have been virtually unchanged for decades and chemotherapeutic drugs used to treat GBM are limited. Arsenic derivatives, known as highly effective anticancer agents for leukemia therapy, has been demonstrated to exhibit cytocidal effects toward GBM cells by inducing cell death, cell cycle arrest, inhibition of migration/invasion, and angiogenesis. Differentiation induction of glioma stem-like cells (GSCs) and inhibition of neurosphere formation have also been attributed to the cytotoxicity of arsenic derivatives. Intriguingly, similar cytotoxic effects against GBM cells and GSCs have also been observed in natural agents such as anthocyanidins, tetrandrine, and bufadienolides. In the current review, we highlight the available data on the molecular mechanisms underlying the multifaceted anticancer activity of arsenic compounds and natural agents against cancer cells, especially focusing on GBM cells and GCSs. We also outline possible strategies for developing anticancer therapy by combining natural agents and arsenic compounds, as well as temozolomide, an alkylating agent used to treat GBM, in terms of improvement of chemotherapy sensitivity and minimization of side effects.

## 1. Introduction

Glioblastoma (GBM) is the most common and lethal intracranial tumor driven from glial cells and characterized by high angiogenic and infiltrative capacities [1,2]. The current standard treatment of GBM consists of maximal surgical resection followed by radiotherapy with concomitant and adjuvant chemotherapy [1,3]. Despite advances in the understanding of the molecular events responsible for disease development and progression, the 5-year survival rate of treated GBM remains <5% [3]. The median survival time for patients is only 14.6 to 20.9 months [4,5].

Temozolomide (TMZ), an oral alkylating chemotherapeutic agent that damages DNA mainly by methylating the O^6^-position of guanine and causing mismatches with thymine in double-strand DNA, has been widely applied as an effective first-line drug for the treatment of newly diagnosed GBM patients [1,2,3,4]. Since GBM is extremely heterogeneous, with genetic, differentiation state, and microenvironmental differences, it is quite common for the lethal tumor to develop resistance to TMZ [6,7]. It has become clear that O^6^-methylguanine DNA methyltransferase (MGMT), capable of reversing guanine methylation that causes DNA damage, is responsible for resistance to TMZ [4,5]. Emerging evidence has also revealed that cancer stem-like cells (CSLCs) of GBM are considered to contribute to drug resistance and tumor recurrence due to their ability for self-renewal and invasion into neighboring tissues [6,7]. In addition, a few key molecular pathways, including PI3K/Akt and Wnt/β-catenin, as well as autophagy, have been linked to TMZ resistance [6,7]. To solve these serious issues, combination therapy is now widely advocated for clinical use and shows a beneficial effect on patient satisfaction [8,9]. Novel therapeutic strategies for combating GBM using TMZ in combination with new promising antitumor drugs have been challenged to improve the overall response rate of GBM and reduce the drug resistance [10,11,12,13,14,15,16,17,18]. Despite this, there is still an urgent need to develop new promising drug combinations options to fight against GBM.

Arsenic and its compounds are widely distributed in the environment and exist in organic and inorganic forms [19]. Arsenic has been used medicinally for over 2000 years around the world. Administration of trivalent arsenic derivatives (arsenite, As^III^) such as arsenic trioxide (As_2_O_3_) has demonstrated superior therapeutic efficacy for acute promyelocytic leukemia (APL) patients [19,20,21]. Several research groups, including us, have conducted detailed analysis of the metabolites of As_2_O_3_ in APL patients [20,21,22,23,24]. Studies on the speciation of As_2_O_3_ metabolites in cerebrospinal fluid (CSF) samples from APL patients treated with As_2_O_3_ alone or combined with mannitol demonstrated that both inorganic arsenic and methylated metabolites exist in CSF, indicating that As^III^ is capable of penetrating into the blood–brain barrier (BBB) [23,24]. Previous studies have shown that As^III^ is efficacious toward GBM cells [25,26,27,28,29], raising the possibility of repositioning arsenic compounds to treat patients with GBM. Intriguingly, darinaparsin, an organic arsenical approved for the treatment of peripheral T-cell lymphoma in Japan [30,31], has been demonstrated to be a more potent cytotoxicity inducer than As_2_O_3_ in various malignant cell lines, including As_2_O_3_-resistant cancer cells [32,33,34], although its cytotoxic effect against GBM cells has not yet been investigated.

Natural agent-derived substances have been being attracted great attention by virtue of their chemopreventive, antitumoral, radiosensibilizing, and chemosensibilizing activities against diverse types of cancers [8,9,19,35,36]. Intriguingly, along with polyphenolic compounds including flavonoids and curcumin [8,37,38], tetrandrine, a bis-benzylisoquinoline alkaloid [39,40], and active bufadienolide compounds [41,42,43,44] have been demonstrated to exhibit potent cytotoxicity toward glioma cells. More importantly, a growing body of evidence has emerged demonstrating anticancer activity of combination treatment of conventional chemotherapeutic drugs and natural agents [8,19,35,40,45,46,47].

In this review, we highlight the available data on the molecular mechanisms underlying the multifaceted anticancer activity of arsenic compounds and natural agents against cancer cells, especially focusing on GBM cells. We further outline possible strategies for developing anticancer therapy by combining natural products and arsenic compounds, as well as TMZ, in terms of the improvement of chemotherapy sensitivity and minimization of side effects.

## 2. Antitumor Activity of Arsenic Compounds

### 2.1. Arsenic Compounds

**Figure 1:**
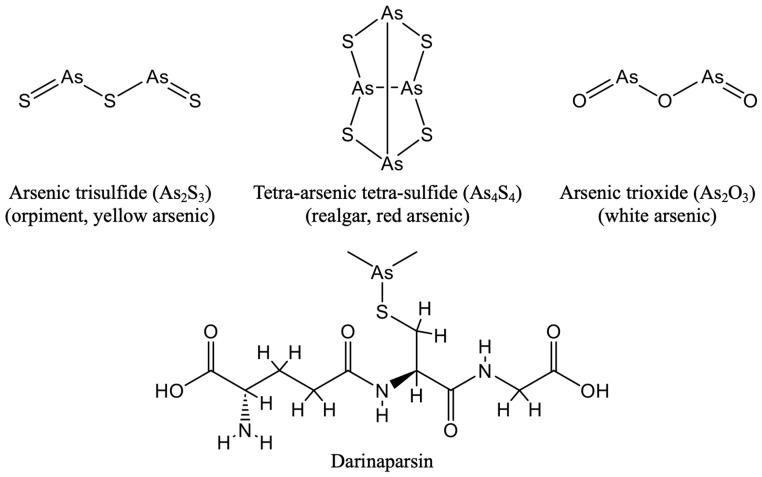
Structural formula of different arsenic compounds.

Despite being a well-known poison and classified as a known human carcinogen, several arsenic compounds have been formulated to treat different diseases including cancer [19,48]. There are three inorganic forms of arsenic: yellow arsenic (As_2_S_3_, also known as orpiment); red arsenic (As_2_S_2_ or As_4_S_4_, also known as realgar); and white arsenic (As_2_O_3_). As mentioned above, darinaparsin, a novel organic arsenic, is composed of dimethylated arsenic and glutathione (GSH) [30,31] (Figure 1).

### 2.2. In Vitro Cytotoxicity and In Vivo Antitumor Activity of Arsenic Compounds

#### 2.2.1. Arsenic Trioxide (As_2_O_3_)

Following a larger-scale clinical evaluation of As_2_O_3_-based regimens against APL worldwide, an impressive effectiveness of the drug led to its approval in the USA and in Europe under the brand name of Trisenox [49,50]. These achievements encourage investigators to explore the detailed mechanisms of action of As_2_O_3_ through biochemical and molecular biological studies. Growing evidence has demonstrated that biomethylation is a major metabolic pathway for inorganic arsenic in human and many animal species [19,51]. Arsenic undergoes metabolic conversion by the reduction of pentavalent arsenate (As^V^) to trivalent arsenic (As^III^) with subsequent methylation, yielding monomethylated and dimethylated metabolites [19,51]. Of note, intermediary metabolites of arsenic, including MAs^III^ (methylarsonous acid) and DMAs^III^ (dimethylarsinous acid), also possess cytocidal effects against different cell types such as leukemia and lymphoma cells, although biomethylation has been considered as a major detoxification pathway for inorganic arsenicals [19,52].

It has been suggested that aquaporin 9 (AQP9), a member of the aquaporin superfamily, is responsible for As^III^ uptake into leukemia cells and has become a promising target in the development of treatment strategies against APL [19,51]. On the other hand, multidrug resistance-associated proteins (MRP1/MRP2), which belong to the ATP-binding cassette transporter superfamily, primarily contribute to the efflux of arsenic, following the formation of arsenic triglutathione, a complex in which As^III^ is bound to the thiol moieties of the cysteinyl residues of three GSH molecules [19,51]. Several research groups, including us, have conducted detailed pharmacokinetic studies of As_2_O_3_ in APL to optimize its treatment [20,21,22]. Previous clinical data have demonstrated that As_2_O_3_ has an ability to cross the BBB in humans regardless of oral or intravenous administration [53,54,55]. We previously conducted a study on speciation of As_2_O_3_ metabolites in CSF samples from APL patients, and showed for the first time that both inorganic arsenic and methylated metabolites existed in CSF, indicating that As^III^ is capable of penetrating into the BBB [23]. These findings thus suggest that As_2_O_3_, with the capability to cross the BBB, can be used for the treatment of patients with central nervous system (CNS) relapse of APL. Fortunately, a non-invasive method via concomitant with 20% mannitol intravenous bolus injection to help As_2_O_3_ enter the CNS has been developed [24,55,56]. Additionally, previous studies have demonstrated that As^III^ exhibits cytotoxicity toward GBM cells [25,26,27]. Collectively, these achievements not only provide meaningful clinical benefits for patients with CNS relapse of APL, but also raise the possibility of repositioning arsenic compounds to treat patients with brain cancer such as GBM.

The mechanisms underlying the anticancer activity of As_2_O_3_ against leukemia have been summarized in several comprehensive reviews [19,50,57,58,59]. In this review, we focused on the mechanisms of action of As_2_O_3_ in advanced solid tumors, especially GBM.

##### Involvement of Oxidative Stress in As_2_O_3_-Mediated Cell Death Induction

Accumulating evidence has shown that oxidative damage largely contributes to As_2_O_3_-mediated cell death induction [19,60]. It has become clear that As_2_O_3_ primarily targets mitochondria, the major source of reactive oxygen species (ROS) generation, and causes a disruption of mitochondrial respiration to elevate the generation of ROS, in which mitoferrin-2 transporter, responsible for maintaining the levels of cellular mitochondrial iron, is involved [61,62,63]. Sun et al. have demonstrated that As_2_O_3_ induces apoptosis-related growth inhibition in rat C6 and 9L glioma cells, whereas only limited effects are observed in normal glial cells [61]. They further clarified that apoptosis induction was closely linked to As_2_O_3_-mediated mitochondria damage along with ROS production [61]. Oxidative stress generally activates nuclear transcription factor E2-related factor 2 (Nrf2) and its downstream target, heme oxygenase-1 (HO-1), and the activation of the Nrf2/HO-1 signaling pathway has been demonstrated to play an important role in the resistance to As_2_O_3_ in leukemic cells [51,64]. In this regard, it has been clarified that HO-1 inhibition or Nrf2 knockdown significantly potentiates the capacity of As_2_O_3_ to induce apoptosis associated with mitochondria damage along with ROS production in a human GBM cell line, U-251 [65]. Haga et al. [62] have also demonstrated that As_2_O_3_ induces ROS production, mitochondrial aggregation, Bax oligomerization, and loss of mitochondrial membrane potential, ultimately leading to apoptosis induction in a human GBM cell line A172, which has the wild-type p53 and low MGMT activity [66,67]. However, similar mitochondrial aggregation and apoptosis induction were not observed in T98G [62], which is a p53 mutant with high levels of MGMT activity [66,67], suggesting that the differences in their sensitivity to As_2_O_3_ may be derived from the different p53 types. In contrast, previous studies demonstrated that As_2_O_3_ induced apoptosis in two GBM cell lines with different p53 status (T98G-mutated; U-87-wt), and further showed that T98G cells were more sensitive to As_2_O_3_ in comparison to U-87 cells [27,68], suggesting the usability of As_2_O_3_ as a novel anti-glioma drug despite differential p53 status. Interestingly, As_2_O_3_ has been recently reported to act as cysteine-reactive compounds that help stabilize the structural mutation of p53 and restore its cancer suppressive activity [69,70]. Given that p53 is the most frequently mutated gene in human cancer and plays crucial roles in regulating the cell cycle, tumor microenvironment, and cell death, including apoptosis, ferroptosis, and autophagy, developing a possible strategy composed of As_2_O_3_ and conventional anticancer drugs may provide novel insights into approaches designed to combat GBM. Mitochondrial glutaminase, encoded by the *GLS* and *GLS2* genes, plays a critical role in cancer cell metabolism linked to redox balance and biosynthesis, and both *GLS* silencing and *GLS2* overexpression synergized with As_2_O_3_ to inhibit malignant properties of glioma cells, including proliferation and migration [71].

An important role of the reduced nicotinamide adenine dinucleotide phosphate (NADPH) oxidase (NOX), another one of the major sources of ROS, has been reported in As_2_O_3_-induced ROS generation and cytotoxicity in leukemia cells such as NB4 cells [72,73]. In comparison, Ludwig et al. recently demonstrated that hypoxic and radiation induced the activity of NOX along with ROS generation, resulting in inactivation of phosphate and tensin homolog (PTEN) and enhancement of Akt phosphorylation, and ultimately promoted survival of PTEN-expressing GBM cells in vitro and in vivo, suggesting that inhibition of NOX activation is a potential therapeutic target in PTEN-functional GBM [74]. These controversial reports indicated differential functional roles of NOX in leukemia and GBM cells, and further suggest that additional studies are warranted to investigate the correlation between the effect of As_2_O_3_ and NOX in PTEN-functional GBM cells. In addition, an interesting report demonstrated that, like ultraviolet irradiation, As_2_O_3_ induced xeroderma pigmentosum group C (XPC) in U-87 cells, and XPC silencing sensitized the cells to As_2_O_3_ via increased oxidative stress [75].

Previous studies have demonstrated that As_2_O_3_ suppresses transcription of the human telomerase reverse transcriptase (hTERT) gene encoding the reverse transcriptase subunit of human telomerase, and ultimately leads to cell death of leukemia cells, in which As_2_O_3_ triggers the oxidation of the Sp1 transcription factor and attenuates its binding to the promoter of hTERT [76,77]. Similarly, As_2_O_3_ induced ROS generation and telomerase dysfunction in glioma cells, resulting in induction of p53- and p21-mediated apoptosis, and G_2_/M cell cycle arrest [25]. The sensitivity of cancer cells to As_2_O_3_ is well known to be dependent on cellular GSH levels, and buthionine sulfoximine (BSO), a GSH depletor, has been demonstrated to act synergistically with As_2_O_3_ in rat C6 glioma cells, suggesting that BSO is suitable as a sensitizing agent with respect to a potential therapeutic use of As_2_O_3_ for the treatment of glioma [78]. X box-binding protein-1 (XBP1) is a transcriptional factor that is activated upon unfolded protein accumulation and is essential for cancer cells to survive under hypoxic conditions [79]. In this regard, Liu et al. demonstrated that XBP1 knockdown significantly enhanced the cell death fraction, mitochondrial membrane potential loss, and ROS levels in As_2_O_3_-treated glioma cells, concomitant with a decrease in several antioxidant molecules including catalase, suggesting targeting XBP1 may have synergistic effects with ROS inducers such as As_2_O_3_ on glioma treatment [80].

##### As_2_O_3_-Mediated Inhibition of Cell Proliferation and Tumor Angiogenesis

Dysregulated cellular processes including cell survival and proliferation, cell cycle arrest, and angiogenesis are linked to the resistance of glioma to conventional anticancer drugs such as TMZ [6,7]. The cell cycle is tightly controlled in a coordinated manner by the cyclin-dependent kinases (CDKs) and their regulatory cyclins (CDK/Cyclin complexes) [81,82]. In this regard, Zhao et al. have demonstrated that As_2_O_3_ induces G_1_ and G_2_ cell cycle arrest in U-87 and T98G GBM cell lines by upregulating the expression of p53 and inhibiting the expression of cyclin B1 [27,68]. Similar G_2_/M arrest and autophagic cell death have also observed in different GBM cell lines following treatment with As_2_O_3_ [25,26]. In agreement, we have clarified that As^III^ triggers cell growth inhibition in U-87 and U-251 cells [47,83]. More importantly, clinically achieved concentrations of As^III^ combined with natural agents exhibited synergistic cytocidal effects on U-87 cells, whereas they showed much less cytotoxicity to human normal peripheral blood mononuclear cells (PBMCs) [47,83]. We also demonstrated that G_2_/M cell cycle arrest was induced by each single agent, and was further strengthened by their combination, and that downregulation of the expression levels of cdc25C, Cyclin B1, and cdc2 was observed in U-87 cells [47,83]. Furthermore, release of enhanced lactate dehydrogenase leakage (LDH), a marker for necrosis, along with downregulation of survivin, was observed in U-87 cells treated with the combined regimen [47,83]. Agents with the capability to trigger necrotic cell death in diverse drug-resistant tumor cells have gained significant attention, since defective or inefficient apoptosis is an acquired hallmark of cancer cells [46,84]. Given a pivotal role of necrosis in overcoming drug resistance of cancer cells, our findings may provide a rationale for the combination regimen of As^III^ plus natural agents. In addition, autophagic cell death was observed in U-87 cells treated with the combined regimen, as evidenced by upregulation of LC3 expression and increment in cell viability by the addition of wortmannin, a potent autophagy inhibitor [47,83].

Intriguingly, our results also showed that SB203580, a specific inhibitor of p38 mitogen-activated protein kinase (MAPK), intensified the cytotoxicity of the combined regimen of As^III^ and natural agent in U-87 cells [47]. Previous findings have shown a pro-survival role for p38 MAPK in GBM cell lines including U-87 cells [41,44,85]. Additionally, phosphorylation of p38 MAPK has been suggested to be a prognostic marker for patients with high-grade glioma, and a combination of vandetanib and a p38MAPK inhibitor may be a useful therapeutic strategy for patients with glioma [86]. Collectively, we hypothesize that combining a p38 MAPK inhibitor with As^III^ may further improve the efficacy of the drug and provide more therapeutic benefits to patients with GBM, although the precise contribution of the p38 MAPK pathway obviously needs to be further investigated.

**Figure 2:**
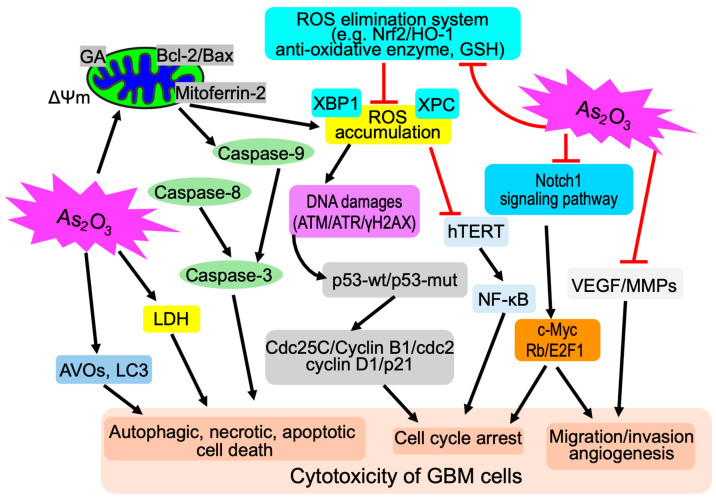
Schematic illustration of the molecular mechanism underlying the pleiotropic anticancer activity of As_2_O_3_ against GBM cells. As_2_O_3_ primarily targets mitochondria by inducing loss of mitochondrial membrane potential (ΔΨm) associated with an imbalance of the Bax/Bcl-2 ratio, as well as upregulation of mitoferrin-2, which in turn causes ROS accumulation, ultimately leads to apoptosis induction and/or cell cycle arrest. Impacts of As_2_O_3_ on ROS elimination system such as anti-oxidative enzymes and GSH also contribute to ROS accumulation, which has been closely linked to DNA damage and inhibition of human telomerase reverse transcriptase (hTERT). Silencing of mitochondrial glutaminase (GA) and xeroderma pigmentosum group C (XPC), inhibition of Nrf2/HO-1, and knockdown of X box-binding protein-1 (XBP1), all of which contribute to maintain the redox balance, sensitize GBM cells to As_2_O_3_. As_2_O_3_ also triggers necrotic and autophagic cell death as evidenced by LDH leakage and restoration of cell viability by the addition of autophagy inhibitors, respectively. Of note, As_2_O_3_ triggers a cytocidal effect against GBM cells regardless of different p53 status (p53-w/p53-mut). Inhibition of the Notch1 signaling pathway and downregulation of VEGF, along with suppression of MMPs (MMP-2 and MMP-9), contribute to As_2_O_3_-mediated cell cycle arrest, inhibition of migration/invasion, and angiogenesis.

In general, the dose of As_2_O_3_ required to suppress solid tumors including GBM is relatively higher than that used to treat APL, which would cause toxicity and undesirable side-effects [60]. Increasing efforts have been proposed to increase the bioavailability of As_2_O_3_ toward solid cancers without increasing its dose, and nanotechnology has emerged as a promising approach to deliver As_2_O_3_ to solid tumors [87,88]. Zhao et al. have demonstrated that intravenous administration of As_2_O_3_ encapsulated in liposomes leads to a 5-fold increase in arsenic concentrations in rat brains in comparison to a single administration of As_2_O_3_, and ultimately induces apoptosis and suppresses tumor angiogenesis via interfering with the expression of vascular endothelial growth factor (VEGF) with low toxicity [89]. In fact, among the known mechanisms of the antitumor activity of As_2_O_3_, the anti-angiogenesis effect is an important characteristic of As_2_O_3_ [90]. In addition to the inhibition of VEGF, downregulation of a series of other key signaling factors implicated in multiple stages of angiogenesis, including the nuclear factor-κB (NF-κB), matrix metalloproteinases (MMPs) such as MMP-2 and MMP-9, and delta-like canonical Notch ligand 4(Dll4)/Notch-1, has been demonstrated to contribute to the anti-angiogenesis effect of As_2_O_3_ in several cancer cell lines derived from various types of tumors, including GBM [88,90,91], although further studies still be needed to provide concrete evidence of the anti-angiogenesis effect of As_2_O_3_ in GBM. The molecular details of the pleiotropic anticancer activity of As_2_O_3_ against GBM cells are proposed and shown in Figure 2.

##### Impact of As_2_O_3_ on Glioma Stem-like Cells (GSCs)

**Figure 3:**
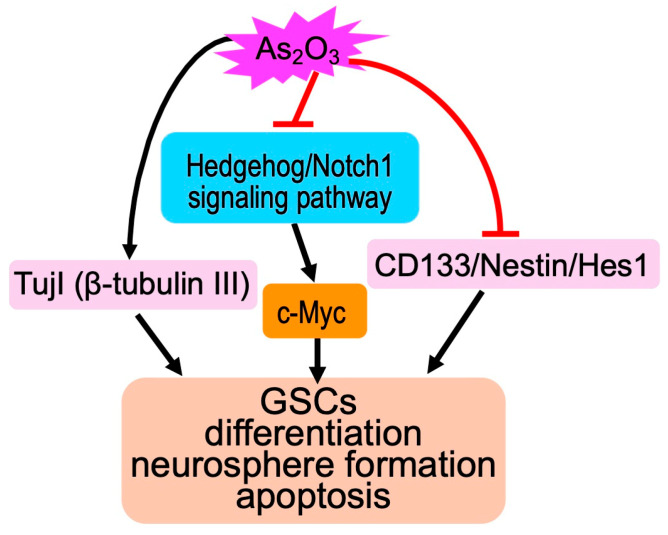
Schematic illustration of the molecular mechanism underlying the pleiotropic anticancer activity of As_2_O_3_ against GSCs. Besides apoptosis induction, As_2_O_3_ suppresses the Hedgehog/Notch1 signaling pathway and downregulates CD133/Nestin/Hes 1, all of which highly express in GSCs, leading to inhibition of neurosphere formation and differentiation of GSCs. In addition, upregulation of TujI (β-tubulin III), a neuronal differentiation marker, is attributed to the differentiation-inducing activity of As_2_O_3_.

GSCs are known to play a crucial role in high resistance against conventional treatments including chemotherapy and radiotherapy, as well as recurrence, which is largely attributed to the activation of Notch and Hedgehog signaling [92,93]. Previous studies have demonstrated that As_2_O_3_ depletes GSCs and inhibits neurosphere recovery and secondary neurosphere formation by downregulation of the Notch pathway [94,95,96]. As_2_O_3_ has also been demonstrated to suppress proliferation and promote apoptosis in three stem-like GBM neurosphere lines by inhibiting both Notch and Hedgehog signaling pathways [97]. Intriguingly, Linder et al. previously demonstrated that As_2_O_3_-mediated Hedgehog/Notch inhibition was capable of synergistically triggering cell death in combination with the natural anticancer agent (*–*)-gossypol (Gos) based on a study using tumor sphere lines and primary patient-derived glioma cultures [98]. In addition, Haydo and colleagues recently demonstrated that GBM tissue infiltration could be effectively blocked through treatment with As_2_O_3_ by using technique combining organotypic tissue culture with light-sheet microscopy (OTCxLSFM) [99]. C-myc, one of several Hedgehog signaling target genes, is known to be required for the maintenance of GSCs and has been closely linked to tumorigenicity and chemoresistance of GBM [100,101]. Yoshimura and colleagues have demonstrated that both As_2_O_3_ and 10058F4, an inhibitor of Myc, induce differentiation of GSCs and that As_2_O_3_ drastically enhances the anti-proliferative effect of 10058F4 [29]. They further showed that treatment of GSC xenografts with As_2_O_3_ and 10058F4 resulted in a significant decrease in tumor growth and increased differentiation concomitant with a decrease in proneural and mesenchymal GSCs in vivo, suggesting a new opportunity for As_2_O_3_ plus 10058F4 as a promising approach for future differentiation therapy of GBM [29]. The molecular details of the pleiotropic anticancer activity of As_2_O_3_ against GSCs are proposed and shown in Figure 3.

#### 2.2.2. Arsenic Disulfide (As_2_S_2_)

As_2_S_2_, another important arsenic compound, is well known for its good therapeutic reputation and perceived low toxicity and has been used to treat several types of hematological disorders, such as chronic myeloid leukemia, acute myeloid leukemia (AML), myelodysplastic syndrome (MDS), and MDS/AML in China [60,102,103,104]. Our previous studies have suggested that the anticancer activities of As_2_S_2_ are attributed to its capability to induce mitochondrial instability, as evidenced by downregulation of Bcl-2, and oxidative stress associated with GSH depletion, which ultimately result in apoptosis and/or differentiation induction in leukemia cells [105,106]. Realgar nanoparticles (NPs) have been designed to improve its bioaccessibility and/or bioavailability. In this regard, a previous report demonstrated that realgar NPs significantly suppressed rat C6 glioma cell proliferation and induced apoptosis by triggering the upregulation of Bax and downregulation of Bcl-2 expression [107]. Intriguingly, Wang and colleagues recently designed high-performance nano-realgar quantum dots (QDs) coupled with 6- aminonicotinamide molecules (NRA QDs) and further encapsulated with a pH-sensitive dextran hydrogel with hyaluronic acid coating (DEX-HA gel) to form a multifunctional nano-realgar hydrogel (NRA@DH Gel) [108]. To evaluate the efficacy of the NRA@DH Gel, they also established in situ mice bearing GL261 brain GBM as animal models assigned to receive an intratumor injection of NRA@DH Gel [108]. Their experimental results demonstrated that NRA@DH Gel triggered the accumulation of ROS by reducing GSH concentrations in tumor cells, suppressed the proliferation and migration of tumor cells, inhibited tumor growth, and extended survival of tumor-bearing mice, endowing a promising future for As_2_S_2_ in the treatment of GBM [108]. The molecular details of the cytotoxicity of As_2_S_2_ against GBM cells are summarized in Table 1.

#### 2.2.3. Darinaparsin

Darinaparsin has been reported to induce apoptosis and G_2_/M cell cycle arrest in tumor cells primarily by triggering mitochondrial dysfunction and caspase activation cascade [30,109], although its antitumor activity against GBM has not yet been investigated in vitro and in vivo. In comparison to As^III^, the molecular mechanisms underlying the uptake and efflux of darinaparsin remain largely uncharacterized. Darinaparsin has been supposed to be transported by x-CT, a major cystine/cysteine cellular importer and coded by the *SLC7A11* gene, following its cleavage by γ-glutamyl transpeptidase and a dipeptidase to ultimately produce dimethylarsino-cysteine [110,111]. Intriguingly, it has been demonstrated that *SLC7A11* overexpression in GBM is associated with increased CSLC properties [112]. Darinaparsin has also been demonstrated to exert its antitumor activity by inhibiting the Hedgehog and/or Notch signaling pathway in various types of cancer cells [30,113]. Given the important roles of *SLC7A11* and Hedgehog/Notch signaling pathways in GBM, studies on the antitumor activity of darinaparsin against GBM are thus warranted. In fact, we recently clarified that darinaparsin exhibited cytotoxic effects on different types of solid cancer cells including the GBM cell line.

## 3. Potential of Natural Products Against GBM

### 3.1. Anthocyanidins

We have previously highlighted the chemopreventive and anticancer activities of polyphenolic compounds based on scientific experimental findings of in vitro and in vivo studies, and further suggested their possibility for clinical use by combining them with conventional chemotherapeutic agents [8,9]. Among these compounds, anthocyanidins are of particular interest.

**Figure 4:**
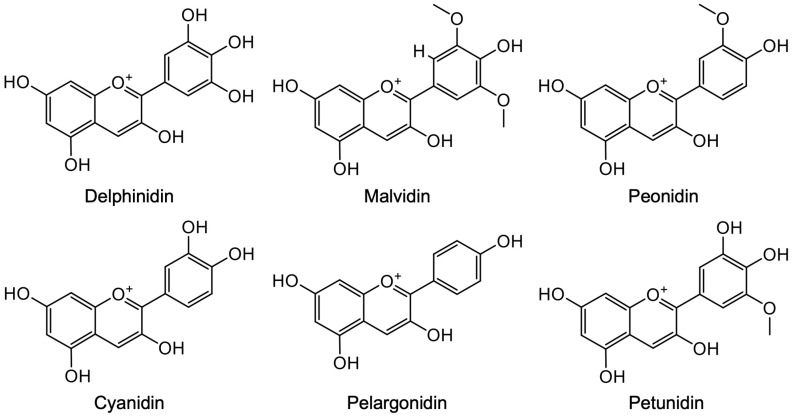
Chemical structures of anthocyanidins.

Anthocyanidins are a diphenylpropane-based polyphenolic ring structure, and are limited to a few structure variants such as cyanidin, delphinidin, malvidin, pelargonidin, peonidin, and petunidin (Figure 4); they present almost exclusively as glycosides and anthocyanins [9,114]. We have previously demonstrated that anthocyanidins, known as one of the flavonoids with a positive charge at the oxygen atom of the C-ring of the basic flavonoid structure, exert cytotoxic effects on the human leukemia cell line HL-60 in the order of delphinidin > malvidin > peonidin > cyanidin > pelargonidin, and suggested that the cytotoxicity of these anthocyanidins might be positively correlated with the presence of hydroxyl groups on ring B of the anthocyanidin molecule [8]. We also provided evidence for the potential combination of As^III^ and delphinidin against NB4 and HL-60 cells, in which delphinidin sensitized these cells to As^III^ by enhancing mitochondrial/death receptor pathway-mediated apoptosis, negatively regulating the amount of intracellular GSH and NF-κB binding activity [36,115]. We further demonstrated that the combination treatment strongly preferred to selectively strengthen the cytotoxicity of As^III^ against cancer cells rather than human PBMCs [36,115]. Intriguingly, Chakrabarti and Ray have demonstrated that delphinidin dose-dependently reduces cell viability in human GBM U-87 and LN18 cells, and that a synergistic cytotoxic effect is observed when combined with 5-Aza-2-deoxycytidine (AzaC), a cytosine nucleoside analog that can cause DNA demethylation [116]. The synergistic cytotoxic effect was attributed to the capability of AzaC to inhibit methylation of the miR-137 promoter region, which was hypermethylated in both GBM cell lines, to trigger an indirect increase in miR-137 expression [116]. They further demonstrated that the combination of miR-137 mimics transfection, and delphinidin treatment achieved similar a synergistic cytotoxic effect in both cells, and revealed that the combined regimen most effectively inhibited not only EGFR, p-Akt, and NF-κB, which are responsible for cell growth and survival, but also VEGF, b-FGF, MMP-9, and MMP-2, which are responsible for angiogenesis and invasion, in both cells [116]. Although delphinidin can cross the BBB [117,118], it remains challenging to increase its bioaccessibility and/or bioavailability. A critical review summarized the delivery of active compounds to brain tissue by using nanoparticle-mediated delivery systems that aimed to optimize delivery of drugs to brain tumors [119]. In this review article, the authors not only highlighted an emerging novel nanomedicine in development for GBM, but also illustrated how mathematical analysis can be utilize to improve the design of delphinidin and related compounds [119]. Delphinidin-3-rutin, a glycoside flavonoid extracted from purple sweet potato, has been demonstrated to repress glioma proliferation both in vitro and in vivo, in which miR-20b-5p/Atg7-dependent cytostatic autophagy is involved [120]. In addition, it has been reported that among anthocyanidins, delphinidin acts as the most potent epithelial–mesenchymal transition (EMT) inhibitor through its inhibitory effect on the transforming growth factor-β (TGF-β)-mediated Smad and extracellular signal-regulated kinase (ERK) signaling pathways, as evidenced by the downregulation of EMT mesenchymal markers fibronectin and Snail, ultimately inhibiting U-87 cell migration [121]. Similarly, a previous report demonstrated that delphinidin, cyanidin, and petunidin significantly suppressed U-87 cell migration by modulating the uPA/uPAR system, known to be associated with invasiveness of various tumors including GBM [122]. All these findings thus provide a rationale for the development of new strategies for GBM therapy. The molecular details of the cytotoxicity of anthocyanidins against GBM cells are summarized in Table 2. For detailed mechanisms underlying the anti-neoplastic potential of natural polyphenolic compounds, including quercetin, luteolin, and curcumin, please refer to some excellent research and review articles [37,38].

### 3.2. Tetrandrine

**Figure 5:**
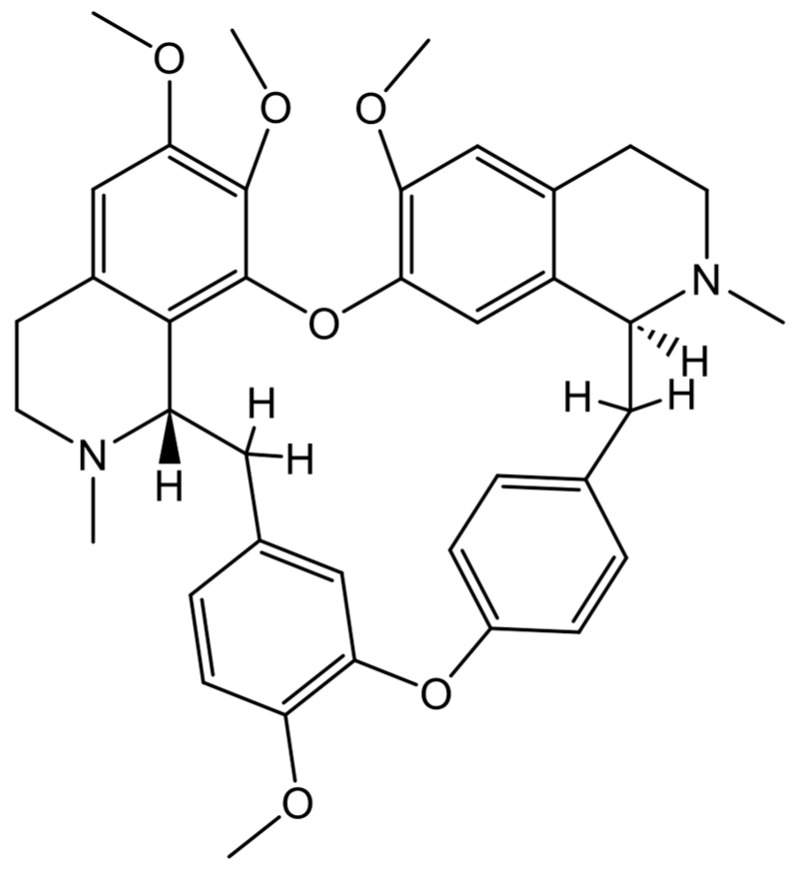
Chemical structure of tetrandrine.

**Figure 6:**
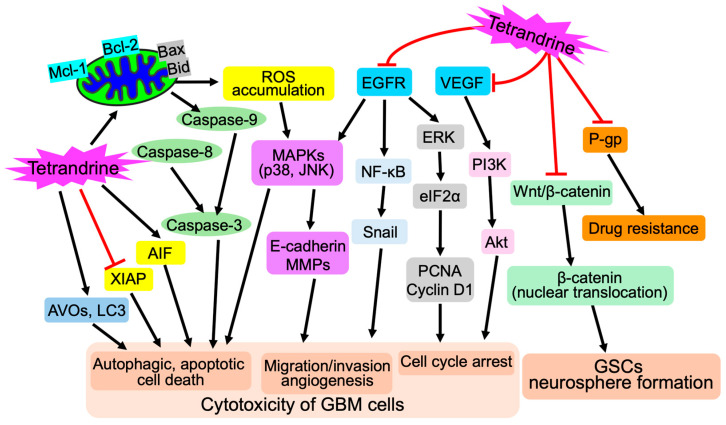
Schematic illustration of the molecular mechanism underlying the pleiotropic anticancer activity of tetrandrine against GBM cells and GSCs. Besides autophagic cell death induction, tetrandrine not only modulates the expression of pro-apoptotic (Bax and Bid) and anti-apoptotic proteins (Bcl-2, Mcl-1, XIAP), but also directly induces apoptosis-inducing factor (AIF), resulting in apoptosis induction. ROS accumulation, because of tetrandrine-meditated mitochondrial dysfunction, can activate MAPKs (p-38 and JNK) and consequently induce cell death. In addition, tetrandrine suppresses EGFR, VEGF, and their downstream targets, resulting in inhibition of migration/invasion and angiogenesis, as well as cell cycle arrest in GBM cells. Tetrandrine also can suppress the Wnt/β-catenin singling pathway, inhibit the nuclear translocation of β-catenin, and ultimately repress neurosphere formation of GSCs. Importantly, tetrandrine has been reported to inhibit a multidrug resistance protein, P-glycoprotein (P-gp), and consequently reverses multidrug resistances, which are closely linked to poor prognosis of GBM.

Tetrandrine, a bis-benzylisoquinoline alkaloid isolated from the root of *Stephania tetrandra* S Moore (Figure 5), has a long history in Chinese medicine for treating different types of diseases such as silicosis, inflammatory pulmonary diseases, and cancer [123]. We have previously demonstrated that tetrandrine induces growth inhibition of different human breast cancer cell lines in a dose-dependent manner, and that the combined regimen of As^III^ and tetrandrine exerts a synergistic cytotoxic effect against these cancer cells by inducing cell cycle arrest, differentiation, and autophagic and apoptotic/necrotic cell death [45,46,124,125]. We further demonstrated the similar synergistic cytotoxic effect against a triple-negative breast cancer cell line MDA-MB-231 in vitro and in vivo, triggered by the combined regimen, suggesting that development of the combination therapy of As^III^ and tetrandrine may provide therapeutic benefit to patients with breast cancer [45]. The anticancer activity of tetrandrine has also been intensively investigated in GBM by virtue of its capability to cross the BBB, since tetrandrine is a highly lipophilic and hydrophobic molecule with a low molecular weight [39]. A comprehensive review of the potential of tetrandrine against gliomas demonstrated that tetrandrine could induce apoptosis, cell cycle arrest, MAPK activation, calcium-activated potassium channel inhibition, oxidative stress, and angiogenesis inhibition [40]. Tetrandrine also could be combined with radiotherapy or other chemotherapeutics to treat glioma by serving as not only a radiosensitizer, but also a multidrug resistance reversing agent [40]. Different research groups have demonstrated that tetrandrine shows substantial anticancer activity against GBM cells in vitro and in vivo by inducing mitochondrial/death receptor pathway-mediated apoptosis and DNA damage-associated cell cycle arrest, and suppressing cell mobility, migration/invasion, and angiogenesis [126,127,128,129,130,131,132]. Given the poor prognosis of GBM patients caused by the obstacle of the BBB and multidrug resistance (MDR), different novel drug delivery systems have thus been explored to overcome these problems. Pang et al. have designed a lactoferrin-conjugated biodegradable polymersome holding doxorubicin and tetrandrine (Lf-PO-Dox/Tet), and demonstrated that Lf-PO-Dox/Tet showed a strong cytotoxic effect toward rat C6 glioma cells and a great uptake index by the cells [133]. By using in vivo models established by stereotactically injecting C6 cells into the right striatum of rats, they further clarified that the tumor volume and the median survival time of the Lf-PO-Dox/Tet group were significantly smaller and longer than that of other therapeutic groups, respectively, suggesting that Lf-PO-Dox/Tet could have therapeutic potential for gliomas [133]. Moreover, multifunctional targeting-drug-loaded liposomes encapsulating vinorelbine and tetrandrine have been constructed and exerted a strong antitumor efficacy both in vitro and in vivo, based on studies using a BBB model, rat C6 glioma cells, GSCs, and glioma-bearing mice [134]. Similar liposomes have been further developed and modified by using transferrin (an iron-binding blood plasma glycoprotein) or RGD (a tripeptide composed of _L_-arginine, glycine, and _L_-aspartic acid), both of which aim to achieve BBB transportation, MDR reversion, and glioma cell targeting simultaneously [135,136]. These modified vincristine/vinorelbine plus tetrandrine liposomes showed a greater ability to improve transport across the BBB, enhance cellular uptake, suppress MDR, and inhibit cancer cell invasion, and ultimately exhibited evident capabilities in diminishing brain glioma in mice [135,136]. In addition to conventional anticancer drugs, a combined regime of tetrandrine and chloroquine, an autophagy inhibitor, also exhibited synergistic antitumor effects in several cancer cell lines, including human GBM cell lines U-87 and U-251, via triggering intracellular ROS accumulation and subsequent activation of the caspase signaling cascade, ultimately leading to cancer cell apoptosis [137]. Intriguingly, Zhang and colleagues demonstrated that tetrandrine inhibited GSCs by repressing the nuclear translocation and expression of β-catenin and triggering mitochondrial pathway-mediated apoptosis [138]. The molecular details of the pleiotropic anticancer activity of tetrandrine against GBM cells and GSCs are proposed and shown in Figure 6.

### 3.3. Bufadienolide Compounds

**Figure 7:**
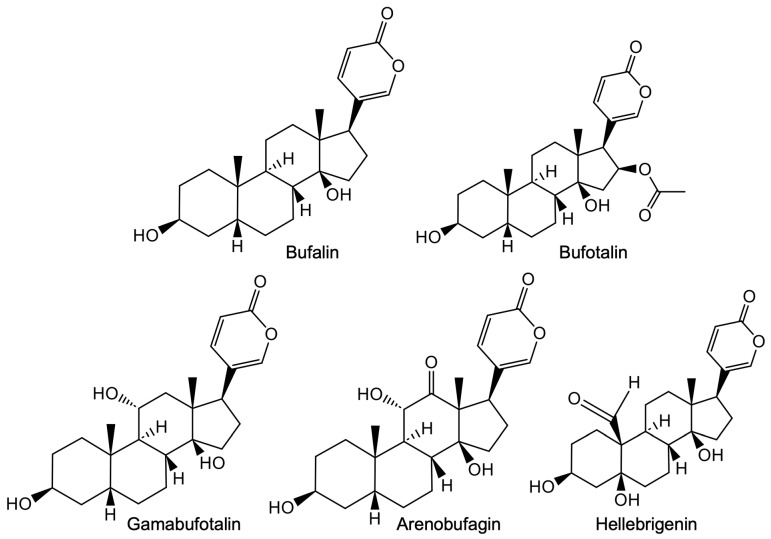
Chemical structures of bufadienolides.

Bufadienolides are the major effective constituents of cinobufacini (also known as Chan Su), a traditional Chinese medicine derived from the dried skin of *Bufo bufo gargarizans* Cantor, and cinobufacini has been used to treat patients with several types of cancers, including hepatoma and lung cancer [139,140,141]. Among active bufadienolide compounds (Figure 7), bufalin appears to be most intensively investigated. It has been demonstrated that bufalin evokes the interplay between apoptosis and autophagy in glioma cells through endoplasmic reticulum stress [142]. Treatment with bufalin resulted in decreased U-251 and U-87 cells viability and G_2_/M arrest, which were attributed in part to impaired mitochondrial and DNA repair functions [143]. In agreement, Li et al. demonstrated that bufalin could promote mitochondrial translocation of Annexin A2 and induce DRP1 (a mitochondrial division protein) oligomers to localize to the surface of mitochondria, which in turn disrupted the mitochondrial division/fusion balance and induced U-251 cell apoptosis [144]. In addition, Lan and colleagues have demonstrated that bufalin inhibits GBM cells growth by expediting proteasomal degradation of the Na^+^/K^+^-ATPase α1 subunit (ATP1A1), known to exert important roles in tumorigenesis of GBM, and apoptosis, both of which are closely linked to the p53 signaling pathway [145,146]. They also showed that bufalin could penetrate the BBB, and that the inhibition of ATP1A1 expression along with tumor growth suppression was observed in xenografted mice [145]. LingHu et al. recently demonstrated that bufalin could cause either apoptosis or necroptosis in human GBM cell lines U-87 and U-373 when caspase-8 was active or inactive, suggesting the ability of bufalin to cause apoptosis and necroptosis might be a good solution for the tumor evasion of apoptosis [42]. Intriguingly, a previous report demonstrated that bufalin inhibited cell proliferation and cancer stem cell-like phenotypes via upregulation of MiR-203 in U-251 and U87 cells [147]. To improve the bioavailability and alleviate the side effects of bufalin, bufalin-loaded PEGylated liposomes (BF/PEG-LP) have been developed, and in vivo study revealed that BF/PEG-LP significantly inhibited the growth of U-251 cells with improved pharmacokinetic and antitumor properties along with lower hemolysis and cytotoxicity [148]. Additionally, we have demonstrated that active bufadienolides, including gamabufotalin, arenobufagin, and hellebrigenin, exhibited selective cytocidal effects against intractable cancer cells such as GBM, but minimal effects on human normal PBMCs [43] and mouse primary astrocytes [41]. It is worthy of note that gamabufotalin and arenobufagin selectively sensitized GBM cells to As^III^ compared to normal cells [47,83]. Our results also suggested that G_2_/M arrest and autophagic and necrotic cell death were attributed to their toxicities, as evidenced by the downregulation of Aurora B, cdc25A, cdc25C, cdc2, Cyclin B1, and survivin, upregulation of p21, an increment in LDH leakage, and increased cell viability caused by the addition of wortmannin in U-87 cells [41,44]. Gamabufotalin also suppressed the expression level of uPA and CA9, and induced the expression level of TIMP3, all of which are closely linked to invasion/metastasis [44]. Of note, we demonstrated that a specific inhibitor of p38 MAPK, SB203580, itself significantly inhibited cell growth, and further strengthened the cytotoxicity of gamabufotalin, arenobufagin, and hellebrigenin in U-87 cells, suggesting a critical pro-survival role for p38 MAPK [41,44]. Given that p38 MAPK serves an important role in promoting GBM cells’ survival, developing a novel combination regimen of these active bufadienolide compounds plus a p38 MAPK inhibitor may enhance the efficacy of these compounds, and may offer substantial therapeutic benefits to patients with GBM. Intriguing, a network pharmacological mechanism of cinobufotalin, derived from the skin of Chinese giant salamander or black sable, against glioma was recently summarized, showing that its efficacy mainly was attributed to induction of cell cycle arrest and apoptosis, as well as regulation of immunity [149]. The molecular details of the cytotoxicity of bufadienolides against GBM cells are summarized in Table 3.

## 4. Possibility of Serving as a Promising Candidate for the Combinatorial Treatment with TMZ

Mechanisms of TMZ resistance in GBM have recently been summarized in several critical reviews [6,7,150]. It has become clear that MGMT repair activity and uniquely resistant populations of GSCs are the most well-known contributors to TMZ resistance [6,7,150]. In addition, a few key molecular pathways, including PI3K/Akt and Wnt/β-catenin, as well as autophagy, have been linked to TMZ resistance [6,7,150]. Herein, we discuss whether the arsenic compounds and natural agents can serve as promising candidate for the combination with TMZ in terms of overcoming resistance to TMZ.

The Hedgehog signaling pathway and its mediator GLI1 play important roles in the development of CNS during embryogenesis [151,152]. Aberrant activation of the Hedgehog/GLI1 signaling pathway plays a central role in GBM pathogenesis, tumor progression, and chemo- and radioresistance associated with GSCs [153,154]. Of note, GLI1 has been linked to increased expression of MGMT, which contains a putative GLI1-binding site [154]. A previous study has showed a positive correlation between Hedgehog/GLI1 pathway activity and MGMT expression in primary GBM tissues and demonstrated that GLI1 overexpression by pcDNA3.1-GLI1 transfection induces MGMT expression and enhances chemoresistance to TMZ in A172 GBM cells [153]. The study not only demonstrated that inhibiting Hedgehog/GLI1 signaling by cyclopamine, a commonly used Hedgehog pathway inhibitor, reduced MGMT expression and decreased chemoresistance to TMZ in U-251 GBM cells, but also showed that cyclopamine inhibited GLI1 and MGMT expression in the GSCs derived from U-87 cells [153]. In agreement, inhibition of the Hedgehog/GLI1 signaling pathway has been linked to tumor growth suppression in GBM [155]. As_2_O_3_ has been reported to block the Hedgehog/GLI1 pathway by directly binding to the GLI1 protein, leading to suppression of human cancer cell growth and tumor development in mice [156]. In addition, As_2_O_3_ prolonged survival of a clinically relevant spontaneous mouse model of medulloblastoma with activated Hedgehog pathway signaling, suggesting that As_2_O_3_ could serve as a Hedgehog pathway inhibitor acting at the level of GLI1 both in vitro and in vivo [156]. The efficacy of As_2_O_3_ in APL has also been partially attributed to its ability to inhibit the Hedgehog/GLI1 signaling pathway [157]. Collectively, these findings suggest that As_2_O_3_ can serve as a candidate for the combinatorial treatment with TMZ. In fact, a previous report has demonstrated that combined treatment with As_2_O_3_ and TMZ achieved a synergistic effect against GBM cells in vitro and in vivo by triggering DNA damage and apoptosis, as evidenced by upregulation of the expression of cleaved caspase-3 and γH2AX, a DNA damage marker, although Hedgehog/GLI1 pathway activity and MGMT expression were not directly investigated [158]. It has also been reported that following the treatment with bio-fabricated nanodrugs with As_2_O_3_/MnCl_2_, the survival rate of GBM-bearing mice is significantly enhanced, and the rate of recurrence is powerfully limited [159]. In addition, an in vitro study aimed to evaluate the antitumor efficacy of As_2_O_3_ in combination with ionizing radiation; furthermore, TMZ against cultured GSCs isolated from a GBM biopsy demonstrated that As_2_O_3_ sensitized GSCs to chemoradiotherapy by inducing its morphologic differentiation, suggesting that As_2_O_3_ exposure before conventional postoperative chemoradiotherapy for GBM might increase treatment efficacy [160]. A clinical research group has previously conducted phase I and II trials of As_2_O_3_ and TMZ in combination with radiation therapy (RT) for patients with malignant gliomas, and demonstrated that the addition of As_2_O_3_ did not improve overall survival in the GBM patients as compared to historic data, although adding As_2_O_3_ to RT and TMZ is feasible with no increased side effects [161,162]. Notably, Han and colleagues recently conducted phase I/II trials of local interstitial chemotherapy with As_2_O_3_ in patients with newly diagnosed glioma [163]. They showed that the maximum tolerated dose of 1.5 mg of As_2_O_3_ was safe and well tolerated [163]. In addition, their results showed the promising efficacy of As_2_O_3_ in patients with WHO grades 2/3/4 and further demonstrated a better clinical outcome in patients with GBM, with a median overall survival of 13.9 months [163]. More recently, Ryu and colleagues conducted a phase I and pharmacodynamic study of As_2_O_3_ plus radiotherapy in patients with newly diagnosed GBM, and showed that As_2_O_3_ together with standard radiation was well tolerated [164]. They further demonstrated that despite the unknown MGMT and isocitrate dehydrogenase status of the patients, overall survival and progression-free survival time was significantly extended, especially in patients who received the twice-weekly arsenic treatment arm compared to the weekly arm without use of TMZ [164]. They also showed that the extended survival time was related with reduced tumor blood flow [164]. Collectively, further investigation into the efficacy of the combination of As_2_O_3_ and TMZ as well as RT is warranted.

Among the abovementioned anthocyanidins, cyanidin-3-O-glucoside has been demonstrated to suppress the β-catenin/MGMT pathway by upregulating miR-214-5p, and ultimately enhance the cytocidal effect of TMZ on a TMZ-resistant LN-18/TR glioma cell line with upregulated β-catenin and MGMT [165]. In vivo studies further showed that cyanidin-3-O-glucoside combined with TMZ significantly suppressed the growth of LN-18/TR tumors, although TMZ had no obvious growth inhibitory effect, providing theoretical support for the clinical treatment of glioma [165].

Regarding bufadienolides, bufalin has been demonstrated to upregulate the expression of the cleaved caspase 3 and poly(ADP-ribose) polymerase and downregulate the expression of human telomerase reverse transcriptase, leading to apoptosis induction in GSCs derived from U-87 and LN-229 GBM cells [166]. More important, bufalin also strengthened the inhibitory effect of TMZ on GSCs by triggering the mitochondrial apoptotic pathway, suggesting that bufalin could damage GSCs, induce apoptosis, and enhance the sensitivity of GSCs to TMZ [166]. These findings thus provide a novel therapeutic approach for patients with glioma in the future.

ATP1A3, a Na^+^/K^+^-ATPase α3 subunit, has also been reported to exhibit potent effects in different types of cancers, including GBM [167]. AQP4, a major aquaporin in the CNS, has been indicated to play important roles in the malignant growth of gliomas [168] and maintaining BBB integrity [169]. In this regard, Lan and colleagues previously clarified a negative feedback loop between ATP1A3 and AQP4 through which gamabufotalin inhibited GBM growth and mediated the synergistic effect of gamabufotalin and TMZ in vitro and in vivo [170]. More recently, Zhu et al. demonstrated that bufotalin possessed an ability to inhibit EMT and trigger apoptosis in GBM cells by inducing mitochondrial dysfunction and decreasing the phosphorylation of Akt via promoting ROS production [171]. They further clarified that bufotalin could significantly augment the chemosensitivity of GBM cells to TMZ in vitro and in vivo, offering potential value for the treatment of GBM patients [171]. The molecular details of the cytotoxicity of bufadienolides against GBM cells and GSCs are summarized in Table 4.

Previous in vitro and in vivo studies have suggested that constituents of Chan Su can boost the host immune system by stimulating the activation of immunocytes [172,173,174]. In agreement, we clarified that nearly non-toxic concentrations of gamabufotalin on PBMCs effectively downregulated the percentages of T-regulatory cells without impacting the percentages of CD4^+^ T cells [43], further suggesting that this compound could be developed as a novel immunotherapeutic agent to combine with TMZ against GBM.

Given the substantial anticancer activity of tetrandrine against GBM cells, and its capability to cross the BBB and act as a radiosensitizer, as well as a multidrug resistance reversing agent, we propose that tetrandrine can serve as a promising candidate in combination with TMZ in terms of overcoming resistance to TMZ, although the efficacy of tetrandrine combined with TMZ obviously needs to be clarified in vitro and in vivo.

## 5. Conclusions

Although tremendous advances have been achieved in understanding the prognosis, treatment response, and treatment targets, there is still an urgent need to develop novel therapeutic strategies for GBM patient treatment. New promising combined regimens of antitumor drugs and TMZ have been challenged in vitro and in vivo to improve the overall response rate of GBM and reduce drug resistance. Recent drug delivery systems formulated using advanced technology such as NPs, QDs, and biodegradable polymers have been applied to accelerate systemic drug delivery to the specific target site, maximizing therapeutic efficacy and minimizing off-target accumulation in the body. Despite this, more preclinical and clinical studies should be conducted to elucidate the efficacy of these promising combined regimens in the treatment of GBM.

## Figures and Tables

**Table 1:** The molecular details of the cytotoxicity of As_2_S_2_ against GBM cells.

Compounds	Cell Lines	Mechanism of Action (Cytotoxicity Profiling)	Type of Study	Ref.
Realgar nanoparticles (As_2_S_2_)	C6	Upregulation of Bax and downregulation of Bcl-2, increment of G_2_/M cell population (apoptosis induction, G_2_/M arrest)	In vitro	[107]
NRA@DH Gel (As_2_S_2_)	GL261	Increment of ROS production along with reduction in GSH concentrations, suppression of colony formation, loss of mitochondrial membrane potential, upregulation of γ-H2AX, suppression of tumor size in vivo (growth inhibition, inhibition of migration/invasion, and tumor formation in vivo)	In vitro, in vivo	[108]

Abbreviations: NRA@DH Gel: a multifunctional nano-realgar hydrogel, ROS: reactive oxygen species.

**Table 2:** The molecular details of the cytotoxicity of anthocyanidins against GBM cells.

Compounds	Cell Lines	Mechanism of Action (Cytotoxicity Profiling)	Type of Study	Ref.
Delphinidin+MiR-137 mimics	U-87, LN18	Inhibition of p-Akt, NF-κB, VEGF, b-FGF, EGFR, MMP-9, MMP-2, activation of caspase-3, -8, 9 (inhibition of migration/invasion, apoptosis induction)	In vitro	[116]
Delphinidin-3-rutin	U-251, A172	Suppression of tumor volume/weight in vivo, downregulation of miR-20b-5p, upregulation of Atg7, LC3 (autophagy induction, inhibition of tumor formation in vivo)	In vitro, in vivo	[120]
Delphinidin, cyanidin, malvidin, pelargonidin, petunidin	U-87	Downregulation of fibronectin and Snail, inhibition of Smad and ERK phosphorylation (inhibition of migration/invasion)	In vitro	[121]
Delphinidin, cyanidin, petunidin	U-87	Suppression of uPA/uPAR (inhibition of migration/invasion)	In vitro	[122]

Abbreviations: b-FGF, basic fibroblast growth factor; EGFR, epidermal growth factor receptor; ERK: extracellular signal-regulated kinase; MMPs: matrix metalloproteinases; NF-κB: nuclear factor-κB; p-Akt: phosphorylation of Akt; Smad: suppressor of mothers against decapentaplegic; uPA, urokinase type plasminogen activator; uPAR: uPA receptor; VEGF: vascular endothelial growth factor.

**Table 3:** The molecular details of the cytotoxicity of bufadienolides against GBM cells.

Compounds	Cell Lines	Mechanism of Action (Cytotoxicity Profiling)	Type of Study	Ref.
Bufalin	U-87, LN229	Upregulation of the ratio Bax/Bcl-2, accumulation of AVOs, upregulation of CHOP, GRP78, LC3, activation of caspase-3, 4 (apoptosis and autophagy induction, endoplasmic reticulum stress)	In vitro	[142]
Bufalin	U-87, U-251	Loss of mitochondrial membrane potential, reduction in ATP production, upregulation of γ-H2AX (G_2_/M arrest, inhibition of migration/invasion)	In vitro	[143]
Bufalin	U-251	Loss of mitochondrial membrane potential, increment of ROS production, activation of p53, caspase-3 (apoptosis induction, S arrest)	In vitro	[144]
Bufalin	U-87, U-251, LN229	Suppression of ATP1A1, induction of degradation of ATP1A1, suppression of tumor volume in vivo, existence of bufalin in CSF (growth inhibition, inhibition of tumor formation in vivo)	In vitro, in vivo	[145]
Bufalin	U-87, U-251, LN229, A172, U118	Loss of mitochondrial membrane potential, increment of ROS production, upregulation of nuclear p53, γ-H2AX, Bax, downregulation of ATP1A1, Bcl-2, activation of caspase-3, -9 (suppression of tumor volume/weight in vivo (apoptosis induction, inhibition of tumor formation in vivo)	In vitro, in vivo	[146]
Bufalin	U-87, U-373	Upregulation of TNFα, TNF receptor 1, RIPK1, induction of necrosome formation (apoptosis and necroptosis induction)	In vitro	[42]
Bufalin	U-87, U-251	Upregulation of miR203, downregulation of Oct4 and Sox2, inhibition of colony formation, increase in Annexin V-positive cells (apoptosis induction, inhibition of neurosphere formation)	In vitro	[147]
BF/PEG-LP	U-251	Increased drug distribution in the brain, suppression of tumor weight in vivo (growth inhibition, inhibition of tumor formation in vivo)	In vitro, in vivo	[148]
Arenobufagin, hellebrigenin	U-87	Existence of arenobufagin in CSF, inhibition of colony formation, downregulation of Cdc25C, Cyclin B1, and survivin, increased LDH leakage, enhanced cytotoxicity by p38 inhibitor (necrosis induction, G_2_/M arrest)	In vitro, in vivo	[41]
Gamabufotalin, arenobufagin	U-87, U251	Downregulation of Cdc25C, Cyclin B1, cdc2 and survivin, uPA, CA9, increased LDH leakage, enhanced cytotoxicity by p38 inhibitor, upregulation of TIMP3 and LC3 (G_2_/M arrest, autophagy and necrosis induction, inhibition of migration/invasion)	In vitro	[44,47,83]

Abbreviations: ATP1A1: Na^+^/K^+^-ATPase α1 subunit; AVOs: acidic vesicular organelles; BF/PEG-LP: bufalin-loaded PEGylated liposomes; CA9, carbonic anhydrase IX; CHOP: C/EBP homologous protein; CSF: cerebrospinal fluid; LDH: lactate dehydrogenase leakage; Oct-4: octamer-binding transcription factor 4; RIPK1: receptor-interacting protein 1; ROS: reactive oxygen species; SOX2: SRY-box transcription factor 2; TNFα: tumor necrosis factor-alpha; TNFR1: TNF receptor 1; TIMP3, tissue inhibitors of metalloproteinase 3; uPA, urokinase type plasminogen activator.

**Table 4:** The molecular details of the cytotoxicity of the combination of arsenic compound/natural products with TMZ against GBM cells and GSCs.

Compounds	Cell Lines/Primary Tumor Cultures	Mechanism of Action (Cytotoxicity Profiling)	Type of Study	Ref.
As_2_O_3_+TMZ	U-87, U-251, U138	Synergistic effect of As_2_O_3_ and TMZ, upregulation of γ-H2AX, activation of caspase-3, suppression of tumor volume in vivo (apoptosis, inhibition of tumor formation in vivo)	In vitro, in vivo	[158]
As_2_O_3_+TMZ+RT	Patient derived GBM GSCs	Suppression of cell viability (growth inhibition)	In vitro	[160]
C3G+TMZ	LN-18/TR (TMZ resistant cells)	Upregulation of miR-214-5p, downregulation of β-catenin, MGMT, upregulation of Annexin V-positive cells, suppression of tumor volume/weight in vivo (apoptosis, inhibition of tumor formation in vivo)	In vitro, in vivo	[165]
Bufalin+TMZ	U-87, LN229, GSCs derived from U-87 and LN229	Synergistic effect of bufalin and TMZ, suppression of colony formation, activation of caspase-3, -9, downregulation of Bcl-2, Bcl-xL, inhibition of telomerase activity (apoptosis induction, inhibition of neurosphere formation)	In vitro	[166]
Gamabufotalin+TMZ	U-87, U-251, LN229, LN18, A172, T98G	Synergistic effect of gamabufotalin and TMZ, upregulation of Annexin V-positive cells, upregulation of ATP1A3 along with inhibition of AQP4, suppression of tumor volume/weight in vivo, increase in survival time (apoptosis induction, inhibition of tumor formation in vivo)	In vitro, in vivo	[170]
Bufotalin+TMZ	U-87, U-251	Suppression of colony formation, upregulation of E-cadherin along with downregulation of vimentin, increment of ROS production, upregulation of Bad, downregulation of Bcl-2, p-Akt, activation of caspase-3, suppression of tumor weight in vivo (apoptosis induction, inhibition of tumor formation in vivo)	In vitro, in vivo	[171]

Abbreviations: AQP: aquaporin; As_2_O_3_: arsenic trioxide; ATP1A3: Na^+^/K^+^-ATPase α3 subunit; C3G: cyanidin-3-O-glucoside; MGMT: O^6^-methylguanine DNA methyltransferase; p-Akt: phosphorylation of Akt; ROS: reactive oxygen species; RT: radiation therapy; TMZ: temozolomide.

## Data Availability

All data generated or analyzed during this study are included in this published article.

## Abbreviations

Akt, protein kinase B; AML: acute myeloid leukemia; APL: acute promyelocytic leukemia; AQP: aquaporin; As_2_O_3_: arsenic trioxide; As_2_S_2_: arsenic disulfide; As^III^: trivalent arsenic; ATP1A1: Na^+^/K^+^-ATPase α1 subunit; ATP1A3: Na^+^/K^+^-ATPase α3 subunit; AVOs: acidic vesicular organelles; AzaC: 5-Aza-2-deoxycytidine; b-FGF, basic fibroblast growth factor; BBB: blood–brain barrier; BSO: buthionine sulfoximine; CA9, carbonic anhydrase IX; CDKs: cyclin-dependent kinases; CNS: central nervous system; CSF: cerebrospinal fluid; CSLCs: cancer stem-like cells; EGFR, epidermal growth factor receptor; EMT: epithelial-mesenchymal transition; GA: mitochondrial glutaminase; GBM: glioblastoma; GSCs: glioma stem-like cells; GSH: glutathione; HO-1: heme oxygenase-1; hTERT: human telomerase reverse transcriptase; LDH: lactate dehydrogenase leakage; MAPKs: mitogen-activated protein kinases; MDR: multidrug resistance; MDS: myelodysplastic syndrome; MGMT: O^6^-methylguanine DNA methyltransferase; MMPs: matrix metalloproteinases; MRP1/MRP2: multidrug resistance-associated proteins; NF-κB: nuclear factor-κB; NOX: reduced nicotinamide adenine dinucleotide phosphate (NADPH) oxidase; NPs: nanoparticles, Nrf2: nuclear transcription factor E2-related factor 2; PBMCs: peripheral blood mononuclear cells; PI3K: phosphatidylinositol 3-kinase; PTEN: phosphate and tensin homolog; QDs: quantum dots; ROS: reactive oxygen species; RT: radiation therapy; TIMP3, tissue inhibitors of metalloproteinase 3; TMZ: temozolomide; uPA, urokinase type plasminogen activator; uPAR: uPA receptor; VEGF: vascular endothelial growth factor; XBP1: X box-binding protein-1; XPC: xeroderma pigmentosum group C.
